# MicroRNA-323a-3p Negatively Regulates NEK6 in Colon Adenocarcinoma Cells

**DOI:** 10.1155/2022/7007718

**Published:** 2022-01-19

**Authors:** Zhongshi Hong, Zhichuan Chen, Jianpeng Pan, Zesheng Shi, Chunxiao Wang, Chengzhi Qiu

**Affiliations:** Department of General Surgery, The Second Affiliated Hospital of Fujian Medical University, Quanzhou 362000, Fujian, China

## Abstract

**Objective:**

The activity of NEK6 is enhanced in several cancer cells, including colon adenocarcinoma (COAD) cells. However, there are few reports on the microRNA (miRNA/miR) regulation of NEK6. In this study, we aimed to investigate the effects of miRNAs targeting NEK6 in COAD cells.

**Methods:**

Public data and online analysis sites were used to analyze the expression levels of NEK6 and miR-323a-3p in COAD tissues as well as the relationship between NEK6 or miR-323a-3p levels and survival in patients with COAD and to predict miRNAs targeting NEK6. Real-time polymerase chain reaction and western blotting were performed to determine the levels of NEK6 and miR-323a-3p in COAD cells. The targeting of NEK6 by miR-323a-3p was verified using a dual-luciferase reporter assay. The 3-(4,5-dimethylthiazol-2-yl)-2,5-diphenyltetrazolium bromide assay, 5-ethynyl-2′-deoxyuridine assay, propidium iodide (PI) staining, annexin V-fluorescein isothiocyanate/PI staining, and transwell assay were employed to test the proliferation, apoptosis, migration ability, and invasiveness of COAD cells.

**Results:**

In COAD cells, NEK6 was highly expressed, whereas miR-323a-3p was expressed at low levels and negatively regulated NEK6. Upregulating the level of miR-323a-3p impaired the proliferation, migration, and invasion of COAD cells and promoted apoptosis, whereas supplementing NEK6 alleviated the damage of the proliferation, migration, and invasion of COAD cells caused by miR-323a-3p and inhibited miR-323a-3p-induced apoptosis. These findings indicate that miR-323a-3p regulates the proliferation, migration, invasion, and apoptosis of COAD cells by targeting NEK6.

**Conclusion:**

miR-323a-3p downregulates NEK6 in COAD cells; this provides a novel basis for further understanding the occurrence and development of COAD.

## 1. Introduction

Colorectal cancer (CRC) is the third most commonly diagnosed cancer and the second leading cause of cancer death worldwide in 2020 [1]. However, the incidence of CRC continues to increase every year in many developing countries. The causes of CRC include genetic, environmental, and lifestyle factors. These factors may cause mutations or abnormal expression of certain oncogenes and tumor suppressor genes, leading to the occurrence or development of CRC [2]. However, the underlying mechanism remains to be elucidated. Therefore, it is necessary to further explore the molecular mechanism of CRC occurrence and development, which can also lay the foundation for finding new therapeutic targets and developing novel drugs for CRC.

NEK6 belongs to the never in mitosis A (NIMA)-related kinases family and is a mitogen/threonine kinase with 313 amino acids. The activity of NEK6 plays important roles in mitotic spindle kinetochore fiber formation, metaphase-anaphase transition, cytokinesis, and checkpoint regulation [3, 4]. Inhibition of NEK6 can lead to the termination of mitosis, chromatin spindle defects, and abnormal chromosomal differentiation [5–7]. In recent years, the expression and kinase activity of NEK6 have been reported to be enhanced in several malignant human cancer cells, including liver cancer [8], prostate cancer [9], gastric cancer [10], breast cancer [11], ovarian cancer [12], and retinoblastoma cancer [13]. In addition, NEK6 has also been associated with inflammation-based diseases, such as esophagitis [14] and ulcerative colitis [15]. The expression level of NEK6 in esophagitis tissue is similar to that in esophageal adenocarcinoma, and overexpression of the NEK6 gene increases in proportion to the severity of esophagitis [14]. Some researchers believe that NEK6 is an attractive target for the development of new anticancer drugs [16]. However, the role and regulatory mechanism of NEK6 on the development of colon adenocarcinoma (COAD) are not fully understood.

Currently, an increasing number of studies have indicated that miRNAs and small noncoding RNA molecules have great potential for the cancer treatment. miRNAs expressed in a wide variety of human cancers can regulate posttranscriptional gene expression by binding to the 3′ untranslated region of the target mRNAs and act as oncogenes or tumor suppressors to regulate cell signaling pathways, affecting tumorigenesis and tumor progression [17]. For example, the overexpression of miR-323a-3p in HCT-116 cells inhibits the K-Ras/Erk1/2 and PI3K/Akt signaling pathways, leading to cell cycle arrest and suppression of cell migration [18]. miR-191 promotes cellular viability of estrogen-dependent breast cancer cells by directly suppressing the expression of DAB2 and might play a critical role in estrogen signaling pathway in the development and progression of ER + breast cancer [19]. Although another study has shown that miR-219-5p inhibits the progression of hepatocellular carcinoma by targeting NEK6 [20], there are few reports on the miRNA regulation of NEK6 in COAD. Therefore, this study aimed to explore the effects of miRNAs targeting NEK6 in COAD cells and to provide a basis for developing RNA therapy strategies for COAD.

## 2. Materials and Methods

### 2.1. Bioinformatics Analysis

Data from the Genotype-Tissue Expression (GTEx) and The Cancer Genome Atlas (TCGA) databases were analyzed using Gene Expression Profiling Interactive Analysis 2 and StarBase, respectively. Patients with COAD were divided into two groups for Kaplan–Meier (KM) curve analysis according to the median level of NEK6 or miR-323a-3p. The predicted miRNAs targeting NEK6 in MicroT-CDS, StarBase, miRDB, TargetScan, and TarBase were used to draw a Venn diagram in the R software (The R Foundation, Vienna, Austria). The patients' data were collected from the public database, and due to its retrospective nature, the study was exempted by the database administrators. Thus, the acceptance from the ethical board was not needed.

### 2.2. Cell Culture

SW480, SW620, HCT-8, and HCT-116 colorectal cancer cells and NCM460 normal human colon mucosal epithelial cell lines were obtained from Xiamen Immocell Biotechnology Co., Ltd (Xiamen, Fujian, China. URL: http://immocell.com/html/cn/pc/cn_about.html). The Roswell Park Memorial Institute (RPMI) 1640 medium (catalog number: R8758; Sigma-Aldrich, Shanghai, China) and McCoy's 5a medium (catalog number: M9309; Sigma-Aldrich) with 10% fetal bovine serum (FBS; catalog number: F8318; Sigma-Aldrich) were used to culture HCT-8 and HCT-116 cells (catalog numbers: IM-H099 and IM-H098, respectively; Xiamen Immocell Biotechnology Co., Ltd. (Immocell), Xiamen, China), respectively. Dulbecco's modified Eagle's medium (DMEM; catalog number: D6429; Sigma-Aldrich, Shanghai, China) containing 10% FBS was used for culturing SW480, SW620, and NCM460 cells (catalog numbers: IM-H111, IM-H112, and IM-H445, respectively; Immocell). The cells were cultured in a humidified incubator containing 5% carbon dioxide at 37°C.

### 2.3. Real-Time Polymerase Chain Reaction (RT-PCR)

Cells were lysed with TRIzol reagent (catalog number: T9424; Sigma-Aldrich) to extract total RNA. One microgram of total RNA was reverse-transcribed into complementary DNA (cDNA) using PrimeScript RT Master Mix (catalog number: RR036A; Takara, Beijing, China), and RT-PCR was performed using the cDNA and TB Green Premix Ex Taq II (catalog number: RR820A; Takara). 18S rRNA was used as an internal control. The primers used for RT-PCR are listed in Table 1. The thermocycling conditions were as follows: initial denaturation at 95°C for 30 s, followed by 40 cycles at 95°C for 10 s, 60°C for 30 s, and 72°C for 30 s. The relative gene expression was measured using the 2−ΔΔCt method.

### 2.4. Western Blotting Assay

Proteins in cells were extracted with precooled radioimmunoprecipitation assay buffer (catalog number: P0013C; Beyotime, Shanghai, China), and protein quantification was performed using a BCA protein concentration determination kit (catalog number: P0012S; Beyotime). Electrophoresis was performed on a 10% sodium dodecyl sulfate-polyacrylamide gel, and the proteins were then transferred to polyvinylidene fluoride (PVDF) membranes (catalog number: IPFL00010; Millipore, Shanghai, China). After incubation with 5% skim milk at 25°C for 2 h, the PVDF membranes containing the proteins were incubated with diluted NEK6 antibody (catalog number: 10378-1-AP; dilution rate: 1 : 1000; Proteintech, Wuhan, China) or GAPDH antibody (catalog number: 10494-1-AP; dilution rate: 1 : 5000; Proteintech) at 25°C for 2 h. After washing, the PVDF membranes were incubated with diluted goat anti-rabbit IgG modified with horseradish peroxidase (catalog number: SA00001-2; dilution rate: 1 : 2000; Proteintech) at 25°C for 1 h. After washing, the PVDF membranes were covered with BeyoECL Moon (catalog number: P0018FM; Beyotime) for exposure. Quantification by densitometry was performed using ImageJ 1.52v (NIH, Bethesda, MD, USA). GAPDH was used as an internal control.

### 2.5. Construction of Plasmids

The pmirGLO vector was used to prepare the plasmids expressing the wild-type (WT) or mutant (MUT) NEK6 3′ UTRs, which were named 3′UTR WT and 3′UTR MUT, respectively. The pCDH vector was used to prepare the NEK6 expression plasmid, which was named pCDH-NEK6. DNAMAN 10.0 was used to design the primers for constructing plasmids (Table 2).

### 2.6. Dual-Luciferase Reporter Assay

Plasmid 3′ UTR WT or 3′ UTR MUT was cotransfected into HCT-8 or SW620 cells with the negative control of miR-323a-3p (mimic NC) or miR-323a-3p mimic using the Lipofectamine™ 3000 transfection reagent (catalog number: L3000075; Invitrogen, Shanghai, China) for 48 h according to the manufacturer's instructions. Subsequently, the firefly luciferase and *Renilla* luciferase activity in cells were detected using a dual-luciferase reporter assay system, which was obtained from Promega (catalog number: E1910; Beijing, China).

### 2.7. 3-(4,5-Dimethylthiazol-2-yl)-2,5-diphenyltetrazolium Bromide (MTT) Assay

The miR-323a-3p or mimic NC was cotransfected into HCT-8 or SW620 cells with the pCDH-NEK6 plasmid or pCDH vector for 24 h, and then the cells were seeded into a 96-well plate at a density of 10,000 cells/well. At different time points (0 h, 24 h, 48 h, and 72 h), the cells' viabilities were tested using the MTT assay as previously described [14]. The absorbance was determined using a spectrophotometer at 490 nm wavelength.

### 2.8. 5-Ethynyl-2′-deoxyuridine (EdU) Assay

HCT-8 and SW620 cells, treated as described above, were cultured in 96-well plates at a density of 10,000 cells/well. After 24 h, the cells were incubated in the RPMI 1640 medium or DMEM containing 50 *μ*M EdU (Guangzhou RiboBio Co., Ltd., Guangzhou, China) for 4 h to allow EdU incorporation. Subsequently, the cells were fixed with 4% paraformaldehyde for 15 min and stained for 15 min with a ClickiT EdU Assay kit (Invitrogen/Thermo Fisher Scientific, Inc.) [15]. A fluorescence microscope (MOTIC, Xiamen, China) was used to observe and capture the images. The number of EdU + or DAPI + dots was counted using Image J 1.52v, and then the percentage of EdU + cells was calculated.

### 2.9. Cell Cycle Assay

After transfection with plasmids or mimics, the cells were cultured in 6-well plates for 48 h and then stained with propidium iodide (PI) (catalog number: A211-01; Vazyme, Nanjing, China) as previously described [14]. A flow cytometer NovoCyte 1300 (ACEA, San Diego, CA, USA) was used to analyze the stained cells.

### 2.10. Apoptosis Assay

After transfection, the cells were cultured in 6-well plates for 48 h. Subsequently, an apoptosis detection kit (catalog number: A211-01; Vazyme, Nanjing, China) was used to stain the cells according to the manufacturer's instructions. Apoptosis was detected using flow cytometer NovoCyte 1300 (ACEA, San Diego, CA, USA).

### 2.11. Transwell Assay

After the miR-323a-3p mimic and pCDH-NEK6 plasmid were cotransfected into HCT-8 and SW620 cells for 24 h, transwell assays were performed for invasion and migration using transwell plates (catalog number: 3422; Corning, Corning, NY, USA) with and without Matrigel (catalog number: 356234; BD Biosciences, Sparks, MD, USA), respectively, as previously described [14]. Afterwards, each transwell chamber was removed, washed twice with phosphate-buffered saline (PBS), fixed in the cell fixation solution for 20 min, and stained with crystal violet (0.5%) for 10 min, the upper surface was wiped with a cotton ball, and the cells were observed under a microscope (Motic, Xiamen, China). The number of penetrating cells was counted and used to evaluate the cell invasion ability. The invasion assay was performed in the same manner as the migration test, with an additional precoating with Matrigel in the upper chamber.

### 2.12. Statistical Analysis

The data from the in vitro experiments were analyzed using the SPSS statistics software (version 22.0; IBM SPSS, Armonk, NY, USA). The Mann–Whitney test was performed for the nonparametric data between two groups. Analysis of variance and Tukey's post hoc tests were used to identify the differences among multiple groups. Student's *t*-test (unpaired) was used to compare the differences between two groups for the parametric data. Differences were considered significant when *p* < 0.05.

## 3. Results

### 3.1. Transcription of NEK6 in COAD Tissues Is Upregulated

**Figure 1:**
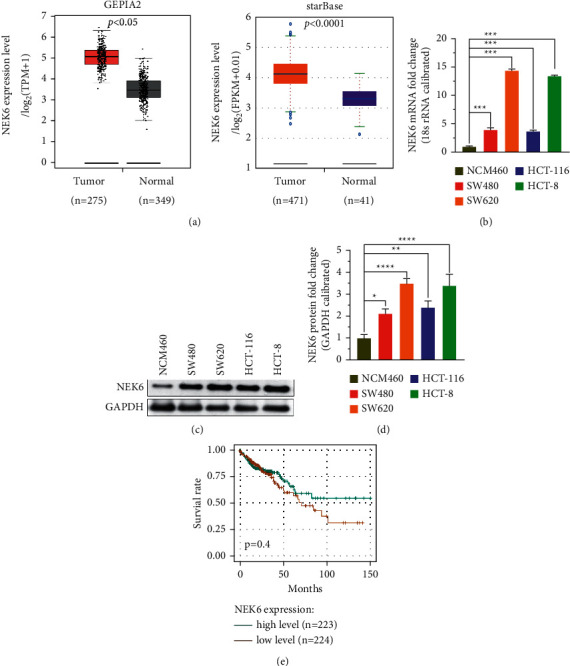
NEK6 was upregulated in COAD tissues and cell lines. (a) The NEK6 mRNA level in normal and tumor tissues from the GTEx and TCGA databases was analyzed using GEPIA2 and StarBase, respectively. (b) NEK6 mRNA level in COAD cell lines and normal colon epithelial cell lines were detected using RT-PCR. (c) Analysis of western blotting showed that NEK6 protein levels in COAD cell lines were higher than those in normal colon epithelial cell lines. (d) Statistical quantification of (c). (e) The relationship between the NEK6 level and patient survival was analyzed using a KM curve. COAD, colon adenocarcinoma. ^*∗*^*p* < 0.05, ^*∗∗*^*p* < 0.01, and ^*∗∗∗*^*p* < 0.001.

To investigate the expression of NEK6 in COAD, we analyzed the data from COAD patients in the TCGA and GTEx databases and found that the NEK6 transcription level in COAD tissues was significantly upregulated (*p* < 0.05; Figure 1(a)). Moreover, by detecting NEK6 levels in different COAD cell lines, we found that the NEK6 transcription and protein levels in COAD cell lines were higher than those in normal colon epithelial cell lines, and the expression level of NEK6 in SW620 and HCT-8 cells was the highest (*p* < 0.05; Figures 1(b)–1(d)). Therefore, subsequent *in vitro* experiments were performed using SW620 and HCT-8 cells. However, the KM curve analysis showed that the expression of NEK6 did not influence the overall survival of COAD patients (*p*=0.04; Figure 1(e)). These results imply that NEK6 is abnormally expressed in COAD tissues.

### 3.2. miR-323a-3p Negatively Regulates NEK6

**Figure 2:**
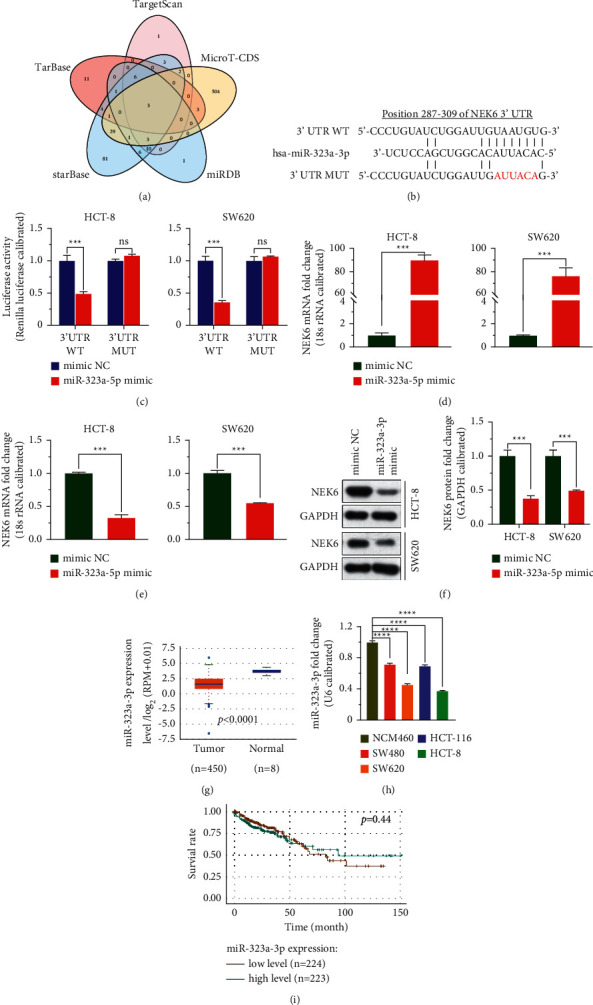
miR-323a-3p negatively regulates NEK6. (a) The miRNAs targeting NEK6 predicted from TargetScan, MicroT-CDS, MiRDB, StarBase, and TarBase were drawn in Venn diagrams. (b) The 3′ UTR of the NEK6 and miR-323a-3p binding sites and the 3′ UTR mutation sites (red) of NEK6 are shown. (c) miR-323a-3p was demonstrated to target the 3′ UTR of NEK6 using a dual-luciferase reporter assay. (d–f) After miR-323a-3p was overexpressed in HCT-8 and SW620 cells, RT-PCR (d, e) and western blotting (f) assays were employed to assess the expression of miR-323a-3p and NEK6. (g) The miR-323a-3p level in COAD tissues from public databases was analyzed. (h) miR-323a-3p levels in COAD cell lines were detected via RT-PCR. (i) The correlation between miR-323a-3p and the overall survival of COAD patients was evaluated via the KM curve. 3′ UTR WT: the wild-type 3′ noncoding region of NEK6; 3′ UTR MUT: the mutant 3′ noncoding region of NEK6; ns: not significant. ^*∗∗∗*^*p* < 0.001 and ^*∗∗∗∗*^*p* < 0.0001.

To study the miRNAs targeting NEK6, the predicted miRNAs were plotted in a Venn diagram. The Venn diagram shows that miR-323a-3p has the potential to target NEK6 (Figure 2(a)). Subsequently, we predicted and mutated the binding sites with miR-323a-3p in the 3′ UTR of NEK6, which was determined using a dual-luciferase reporter assay (*p* < 0.001; Figures 2(b) and 2(c)). Moreover, transfection of the miR-323a-3p mimic into cells upregulated the expression of miR-323a-3p and decreased the expression of NEK6, indicating that miR-323a-3p negatively regulated the expression of NEK6 (*p* < 0.001; Figures 2(d)–2(f)). The results of the StarBase analysis showed that miR-323a-3p expression was significantly decreased in COAD tissues (Figure 2(g)). In addition, *in vitro* experiments indicated that the expression of miR-323a-3p in the COAD cell lines was significantly downregulated and was the lowest in SW620 and HCT-8 cells (*p* < 0.0001; Figure 2(h)). However, the KM curve analysis showed that miR-323a-3p did not affect the overall survival of COAD patients (*p*=0.44; Figure 2(i)). These findings indicate that miR-323a-3p is weakly expressed in COAD tissues and cells and downregulates NEK6 by targeting the 3′ UTR of NEK6.

### 3.3. miR-323a-3p Inhibits Cell Proliferation by Negatively Regulating NEK6

**Figure 3:**
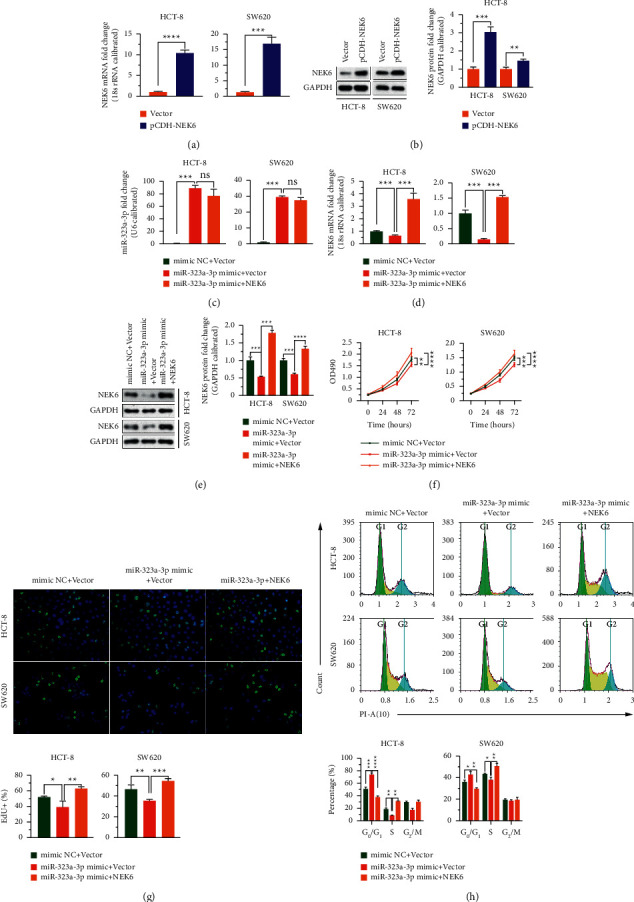
miR-323a-3p inhibits cell proliferation by negatively regulating NEK6. (a, b) After cells were transfected with the pCDH-NEK6 plasmid, the expression level of NEK6 was evaluated via RT-PCR (a) and western blotting (b) assays. (c–h) After the miR-323a-3p or mimic NC was cotransfected into HCT-8 and SW620 cells with the pCDH-NEK6 plasmid or pCDH vector, RT-PCR (c, d) and western blotting (e) assays were performed to assess the expression levels of miR-323a-3p and NEK6. MTT (f) and EdU (g) assays were employed to evaluate cell proliferation, and PI staining was performed for the cell cycle assay (h). PI: propidium iodide; ns: not significant, ^*∗*^*p* < 0.05, ^*∗∗*^*p* < 0.01, ^*∗∗∗*^*p* < 0.001, and ^*∗∗∗∗*^*p* < 0.0001.

To explore whether miR-323a-3p negatively regulates NEK6 and influences the proliferation of COAD cells, we overexpressed both miR-323a-3p and NEK6 in COAD cells. Transfection of pCDH-NEK6 into HCT-8 and SW620 cells significantly increased the expression level of NEK6 in the cells (*p* < 0.001; Figure 3(a) and 3(b)). Transfection of the miR-323a-3p mimic into the cells improved the level of miR-323a-3p and decreased the expression of NEK6 compared to that in cells transfected with the miR-323a-3p mimic alone. The expression of miR-323a-3p in cells cotransfected with the miR-323a-3p mimic and pCDH-NEK6 plasmid did not change significantly, indicating that NEK6 did not regulate miR-323a-3p and that overexpression of miR-323a-3p inhibited the expression of NEK6, whereas supplementing NEK6 alleviated the miR-323a-3p-induced downregulation of NEK6 expression (*p* < 0.001; Figures 3(c)–3(e)). Furthermore, the results of the MTT and EdU assays suggested that upregulation of miR-323a-3p suppressed cell proliferation, whereas supplementing NEK6 alleviated the miR-323a-3p-induced suppression of cell proliferation (*p* < 0.05; Figures 3(f) and 3(g)). Cell cycle analysis showed that increasing miR-323a-3p arrested the cell cycle in the *G*_0_/*G*_1_ phase, whereas replenishing NEK6 retarded miR-323a-3p-induced cell cycle arrest (*p* < 0.05; Figure 3(h)). These data reveal that miR-323a-3p downregulates NEK6 expression to suppress proliferation.

### 3.4. miR-323a-3p Promotes Apoptosis by Downregulating NEK6

**Figure 4:**
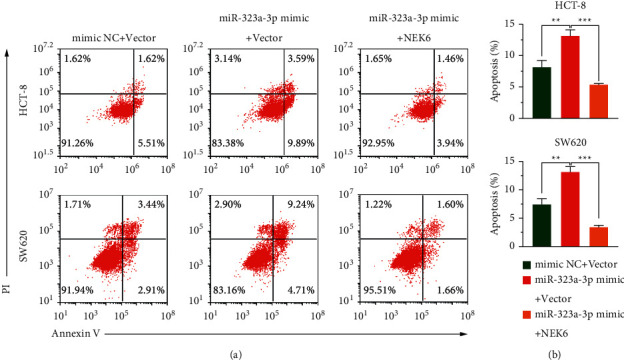
miR-323a-3p promotes apoptosis by downregulating NEK6. (a, b) After miR-323a-3p or mimic NC was cotransfected into HCT-8 and SW620 cells with the pCDH-NEK6 plasmid or pCDH vector, apoptosis was assessed following annexin V-FITC/PI staining (a), and the apoptotic proportion was calculated (b). PI: propidium iodide. ^*∗∗*^*p* < 0.01 and ^*∗∗∗*^*p* < 0.001.

To investigate whether the miR-323a-3p targeting of NEK6 affects COAD cell apoptosis, we detected cell apoptosis. Overexpression of miR-323a-3p in HCT-8 and SW620 cells increased apoptosis, whereas supplementation with NEK6 alleviated the apoptosis induced by miR-323a-3p (*p* < 0.01; Figure 4), indicating that miR-323a-3p induces apoptosis by regulating the expression of NEK6.

### 3.5. miR-323a-3p Damages Migration and Invasion by Regulating NEK6

**Figure 5:**
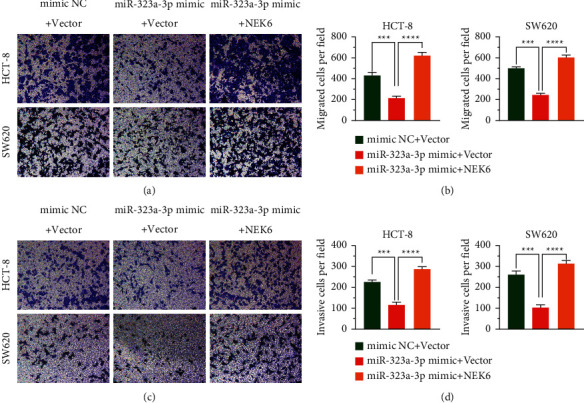
miR-323a-3p inhibits migration and invasion by regulating NEK6. The miR-323a-3p or mimic NC was cotransfected into HCT-8 and SW620 cells with the pCDH-NEK6 plasmid or pCDH vector. (a–d) A transwell assay was performed to examine cell migration (a, b) and invasion (c, d). ^*∗∗∗*^*p* < 0.001 and ^*∗∗∗∗*^*p* < 0.0001.

The migration and invasion of cancer cells to surrounding tissues and vasculature are important steps in cancer metastasis [16]. To explore the role of miR-323a-3p in downregulating NEK6 in the migration and invasion of COAD cells, we performed a transwell assay to assess the migration and invasiveness of cells. The results indicated that upregulation of miR-323a-3p inhibited cell migration and invasion, whereas supplementing NEK6 enhanced the cell migration and invasion capabilities (*p* < 0.001; Figure 5). These data suggest that miR-323a-3p suppresses the migration and invasion of COAD cells by downregulating NEK6.

## 4. Discussion

The *NEK6* gene is located on chromosome 9q33.3 and plays a crucial role in mitotic cell cycle progression. NEK6 phosphorylated by activated NEK9 regulates mitotic spindle formation through the phosphorylation of kinesin Eg5 [21]. It has been reported that NEK6 is involved in the establishment of a microtubule-based mitotic spindle and DNA damage response, which is directly phosphorylated by CHK1 and CHK2, and may be a novel target for DNA damage checkpoints [7, 12, 22, 23]. It is worth noting that NEK6 overexpression is associated with tumorigenesis and cancer progression in several solid tumors [8–11]. NEK6 overexpression also exists in colorectal cancer (CRC) and colorectal adenomatous polyp (CRAP) and was significantly correlated with the large polyp diameter [15]. In this study, we found that NEK6 is highly expressed in COAD cells, promotes proliferation and migration, and inhibits apoptosis, which is consistent with other solid tumor reports. NEK6 overexpression drives tumorigenesis in COAD by involving multiple signaling pathways, including regulation of cyclin B transcription levels mediated by Cdc2 [8], inhibition of TGF-*β* pathway by interfering with nuclear translocation Smad4 [24], and the activation of STAT3 signaling [25, 26].

A large number of studies have shown that miRNAs regulate more than one-third of all human genes in a sequence-specific manner [27–30]. Aberrant regulation of miRNAs functionally promotes the occurrence and development of different human cancers by regulating the degradation or translation of mRNAs [31–34]. Recent studies showed that NEK6 might also exhibit expression modulation through interaction with miRNAs. These miRNAs directly target NEK6 mRNA and downregulate NEK6 expression, including miR-506-3p, miR-219-5p, and miR-141-3p, resulting in inhibition of cell proliferation and induction of apoptosis in retinoblastoma, HCC, and clear cell renal cell carcinoma [13, 20, 35]. In this paper, we confirmed that miR-323a-3p could also negatively regulate NEK6 expression in COAD cells through binding with complementary 3′-UTR sequences in target NEK6 mRNA by luciferase reporter assay and binding site mutation analysis and block the proliferation of colon cancer cells.

miR-323a-3p is located on chromosome 14q32.31 and acts as a tumor suppressor gene in CRC [36]. Furthermore, it has been found that the upregulation of miR-323a-3p expression can induce apoptosis and inhibit the proliferation and migration of glioma cells by targeting IGF-1R [37] and damage the progression of epithelial-mesenchymal transition in bladder cancer cells by regulating the MET/SMAD3/SNAIL circuit [38] and negatively regulate LDHA expression to disrupt glycolysis in osteosarcoma cells [39]. In addition, miR-323a-3p has been associated with depression and lung fibrosis [40, 41]. However, the target genes of miR-323a-3p in COAD cells remain unknown. Our results indicate that miR-323a-3p targets NEK6 mRNA to functionally alter downstream NEK6 expression and arrest subsequently colon cancer cell proliferation. Moreover, the level of miR-323a-3p is downregulated in COAD tissues and cells. Although the KM curve showed that miR-323a-3p did not significantly affect the overall survival of COAD patients, the miR-323a-3p overexpression in COAD cells inhibited cell proliferation, blocked the cell cycle, promoted cell apoptosis, and inhibited migration and invasion, indicating that the overexpression of miR-323a-3p suppressed the malignant quality of COAD cells. The current study revealed that both miR-323a-3p and NEK6 could regulate the cell phenotype by targeting the TGF-*β* signaling pathway in some diseases, including hepatocellular carcinoma [24], pancreatic ductal adenocarcinoma [42], lung fibrosis [40], and cardiac fibrosis [43]. Our results illustrate the crosstalk function of miR-323a-3p and NEK6. However, the effect of miR-323a-3p targeting NEK6 on the TGF-*β* signaling pathway needs to be further studied.

Due to the lack of animal experiments in this study, it is impossible to directly investigate the role of miR-323a-3p targeting NEK6 in COAD, which is a limitation of this study.

## 5. Conclusions

In brief, this study revealed that miR-323a-3p regulates the cell life course (proliferation and apoptosis) and biological processes (migration and invasion) of COAD cells by negatively regulating NEK6, laying a foundation for further understanding the mechanism of COAD occurrence and development.

## Figures and Tables

**Table 1:** The primers for RT-PCR.

Name	Sequence (5′-3′)
NEK6-QF	TGAAGCTCGGTGACCTTGGTCT
NEK6-QR	GTCGGACTTGAAGTTGTAGCCG
miR-323a-5p-RT	GTCGTATCCAGTGCAGGGTCCGAGGTATTCGCACTGGATACGACGCGAAC
miR-323a-5p-QF	AGGTGGTCCGTGGCGC
miR-323a-5p-QR	AGTGCAGGGTCCGAGGTATT

QF: forward primer for RT-PCR; QR: reverse primer for RT-PCR; RT: reverse transcription.

**Table 2:** The primers for construction of plasmids.

Name of plasmids	Name of primers	Sequence (5′-3′)
Wild-type NEK6 3′ UTR	3′UTR WT-F	GAGCTCGCTAGCCTCGAGCCGTGCCTTATCAAAGCCAG
	3′UTR WT-R	GCATGCCTGCAGGTCGACGCAGCAGGTGTCAGGAATC
Mutant NEK6 3′ UTR	3′UTR MUT-F	TGGATTGATTACAGAATCTTTAGGGTAATTC
	3′UTR MUT-R	AAGATTCTGTAATCAATCCAGATACAGGGGGC
pCDH-NEK6	pCDH-Nflag-F	CTAGAGCTAGCGAATTCGCCACCATGGACTACAAAGACGATGACGAC
	Flag-NEK6-F	ACAAAGACGATGACGACAAGATGCCCAGGAGAGAAGTTTG
	pCDH-NEK6-R	CTCAGCGGCCGCGGATCCGGTGCTGGACATCCAGATG

F: forward primer; R: reverse primer.

## Data Availability

The datasets used and/or analyzed during the current study are available from the corresponding author on reasonable request.
